# Conceptions on pharmaceutical services in Brazilian primary health care

**DOI:** 10.11606/S1518-8787.2017051007107

**Published:** 2017-09-22

**Authors:** Ediná Alves Costa, Patrícia Sodré Araújo, Thais Rodrigues Penaforte, Joslene Lacerda Barreto, Augusto Afonso Guerra, Francisco de Assis Acurcio, Ione Aquemi Guibu, Juliana Alvares, Karen Sarmento Costa, Margô Gomes de Oliveira Karnikowski, Orlando Mario Soeiro, Silvana Nair Leite

**Affiliations:** I Instituto de Saúde Coletiva. Universidade Federal da Bahia. Salvador, BA, Brasil; II Departamento de Ciências da Vida. Universidade do Estado da Bahia. Salvador, BA, Brasil; III Departamento do Medicamento. Faculdade de Farmácia. Universidade Federal da Bahia. Salvador, BA, Brasil; IV Departamento de Farmácia Social. Faculdade de Farmácia. Universidade Federal de Minas Gerais. Belo Horizonte, MG, Brasil; V Faculdade de Ciências Médicas. Santa Casa de São Paulo. São Paulo, SP, Brasil; VI Núcleo de Estudos de Políticas Públicas. Programa de Pós-Graduação em Saúde Coletiva. Universidade Estadual de Campinas. Campinas, SP, Brasil; VII Programa de Pós-Graduação em Saúde Coletiva. Departamento de Saúde Coletiva. Faculdade de Ciências Médicas. Universidade Estadual de Campinas. Campinas, SP, Brasil; VIII Programa de Pós-Graduação em Epidemiologia. Faculdade de Medicina. Universidade Federal do Rio Grande do Sul. Porto Alegre, RS, Brasil; IX Faculdade de Ceilândia. Universidade de Brasília. Brasília, DF, Brasil; X Faculdade de Ciências Farmacêuticas. Pontifícia Universidade Católica de Campinas. Campinas, SP, Brasil; XI Departamento de Ciências Farmacêuticas. Universidade Federal de Santa Catarina. Florianópolis, SC, Brasil

**Keywords:** Pharmaceutical Services, Health Knowledge, Attitudes, Practice, Health Personnel, Comprehension, Assistência Farmacêutica, Conhecimentos, Atitudes e Práticas em Saúde, Pessoal de Saúde, Compreensão

## Abstract

**OBJECTIVE:**

To identify and discuss the conceptions of pharmaceutical services in Brazilian Primary Health Care, according to different subjects.

**METHODS:**

This study is part of the *Pesquisa Nacional sobre Acesso, Utilização e Promoção do Uso Racional de Medicamentos – Serviços, 2015* (PNAUM – National Survey on Access, Use and Promotion of Rational Use of Medicines – Services, 2015), which is composed of an information survey in a representative sample of cities, stratified according to Brazilian regions, and a subsample of primary health care services. Municipal secretaries of health, those responsible for pharmaceutical services, and those responsible for medicine delivery in pharmacies/dispensing units of the selected services were interviewed. The questionnaires included one question about the understanding of the interviewee regarding pharmaceutical services. The content analysis technique was used to apprehend, in the statements, the meanings attributed to pharmaceutical services, which were subsequently classified into categories according to their main conceptions.

**RESULTS:**

Among the wide diversity of conceptions on pharmaceutical services (PS), we highlight the ones focused on 1) logistic control of medicines with activities concerning guidance or information on their use and 2) guidance or information to users on the use of medicine. The findings reveal a shifting tendency from a medicine-focused conception to one that considers the users and their needs as the final recipient of these actions. However, the lack of references to conceptions regarding care management and integrality point out the slowness of this change; after all, this is a social and historical process that comprises the production of meanings that transcend legal, logistic, and technical arrangements in pharmaceutical services.

**CONCLUSIONS:**

The diversity of conceptions expresses the several meanings attributed to pharmaceutical services; we also identified, in their reorientation process, a movement that reflects a gradual shift in the technical paradigm, from the focus on medicine logistics to a user-oriented approach of health services.

## RESUMO

**OBJETIVO:**

Identificar e discutir as concepções de assistência farmacêutica segundo distintos atores, na Atenção Primária à Saúde, no Brasil.

**MÉTODOS:**

Estudo integrante da Pesquisa Nacional sobre Acesso, Utilização e Promoção do Uso Racional de Medicamentos – Serviços, 2015, composta por levantamento de informações numa amostra representativa de municípios, estratificada pelas regiões do Brasil, e sub-amostra de serviços de atenção básica. Entrevistaram-se os secretários municipais de saúde, os responsáveis pela assistência farmacêutica e os responsáveis pela entrega de medicamentos nas farmácias/unidades de dispensação dos serviços selecionados. Os questionários incluíram uma pergunta acerca do entendimento dos entrevistados sobre assistência farmacêutica. Utilizou-se a técnica de análise de conteúdo, buscando-se apreender nos enunciados os sentidos atribuídos à assistência farmacêutica, que posteriormente foram classificados em categorias com as principais concepções.

**RESULTADOS:**

Entre a grande diversidade de entendimentos sobre AF destacaram-se as concepções centradas 1) no controle logístico dos medicamentos com atividades de orientação ou informação sobre o uso e 2) na orientação ou informação ao usuário sobre o uso do medicamento. Os achados revelam tendência de deslocamento de uma centralidade no medicamento para uma concepção mais ampliada que inclui o usuário e suas necessidades como o destinatário final dessas ações. Entretanto, a pouca referência a concepções relacionadas à gestão e integralidade da atenção apontam a lentidão da mudança; afinal trata-se de um processo social e histórico que envolve a produção de sentidos que transcendem os arranjos legais, logísticos e técnicos na organização da assistência farmacêutica.

**CONCLUSÕES:**

A diversidade de concepções expressa os muitos sentidos atribuídos à assistência farmacêutica; identificando-se também, no processo de sua reorientação, um movimento que reflete uma mudança gradual do paradigma técnico, centrado na logística de medicamentos, para uma abordagem orientada ao usuário dos serviços de saúde.

## INTRODUCTION

Since its regulation, in 1990, the Brazilian Unified Health System (SUS) has been implementing several strategies to place pharmaceutical services (PS) as an essential public policy to health care integrality as a social right and duty of the State. Efforts show increases in population access to medicines and the need for change in the organization of pharmaceutical services in the Country^a^. Pharmaceutical services have a systemic and multidisciplinary nature that covers multiple actions on the promotion, protection, and recovery of individual and collective health, aiming the access and rational use of medicines^12^ ^,^ ^14^.

According to Wiedenmayer et al.^24^, pharmacy services became more sophisticated by changing some roles and introducing new ones. We can identify changes in professional practice, from a procedure more focused on the medicine to another that emphasizes the shared responsibility between patients and pharmacists, which demands for the accountability of these professionals not only when it comes to the medicine dispensing but also on the needs of individuals.

Traditional roles, which comprise preparation, dispensing, or sale of medicines, are clearly insufficient to characterize PS from an integral perspective regarding actions on health and sectoral policies. In this sense, PS must be established as a set of systematically carried out activities that considers the medicines, but is mainly focused on the patient^12^.

However, in Brazil, the main focus of pharmaceutical work is still limited to the control and distribution of medicines^3^ ^,^ ^21^. Over the past few decades, despite the strong tendency of incorporating new practices, such as pharmaceutical care – which stimulates pharmacists to adopt a more inclusive approach in patient care –, this type of professional practice is still very limited in health services. Hence, to achieve the goals of the *Política Nacional de Assistência Farmacêutica* (PNAF – National Policy of Pharmaceutical Services), there is need to improve the quality of pharmaceutical services, which must not only consider the technical and practical aspects of the job, but also the subjective ones, since the perceptions on PS are essential to change this professional paradigm^24^.

Only after ten years from the initial implementation processes of SUS, the first initiatives to restructure PS were established. Despite being late, subsequent efforts were undertaken in the field to ensure the access and rational use of medicines, having reorientation practices^21^ as a structural axis towards the improvement of management and pharmaceutical services. Thus, PS must result from the combination of structure, people, and technologies, in a certain social context, to improve the quality of life of the population by implementing actions on health promotion, prevention, recovery, and rehabilitation^15^.

The elaboration of a theoretical and conceptual framework for PS represents a challenge, considering the complexity of the field, which comes mainly from its several social life configurations. Synthetically, we point out the focus on medicines in PS as an enhancing factor of this complexity, given that medicines are objects surrounded by science, the market, and society, and that have several meanings^18^. However, in the context of health policies, it is possible to consider that medicine and PS policies are still very recent, contributing to the maintenance of a situation surrounded by old and new ideologies, conceptions, and worldviews on PS.

All this corroborates to the existence of several definitions of PS that comprise different meanings/understandings/conceptions^2^ ^,^ ^3^. However, currently we have examples that show the break of technical standards and the emergence of a more social identity for pharmaceutical services – still very recent and conflicting in its formation^20^. We inferred the importance of discussing PS initiatives by identifying the different perspectives of their key-subjects, this way considering the characteristics that would allow us to develop a representational axis and an experience of professional practice.

This article aims at identifying and discussing the conceptions on PS in Brazilian primary health care according to different subjects. We take conceptions on PS as social representations^11^, understandings, worldviews, common senses, and ideas. This refers to a political exercise regarding the positioning and prioritization of PS by these subjects and in the health system in which they carry out their technical, clinical, political, and administrative practices with the meanings assigned to them.

## METHODS

This study is part of the *Pesquisa Nacional sobre Acesso, Utilização e Promoção do Uso Racional de Medicamentos* – *Serviços, 2015* (PNAUM – National Survey on Access, Use and Promotion of Rational Use of Medicines – Services, 2015), which aimed to characterize the organization of pharmaceutical services in primary health care/SUS – for promoting the access and rational use of medicines –, as well as to identify and discuss the factors that interfere in the consolidation of pharmaceutical services in the cities. PNAUM is a cross-sectional study of exploratory and analytical nature, composed by an information survey in a sample of primary health care services, in representative cities of Brazilian regions. Several study populations were considered in the sampling plan; these samples were stratified according to the regions, which constitute the study domains^1^.

This is a descriptive and exploratory study that aimed to understand PS conceptions from the content analysis of reports given by the interviewees. Interviews were conducted with municipal secretaries of health (MSH), those responsible for pharmaceutical services (RPS) management of the selected cities from different regions, and those responsible for medicine delivery to patients in the pharmacies/dispensing units sampled in the cities. In this study, only pharmacists (PHARM) were selected among those responsible for medicine delivery.

The interviews were carried out with structured questionnaires that included the sociodemographic characteristics of the interviewees and a question on their understanding of PS. Municipal secretaries of health and those responsible for PS management were interviewed by phone and those responsible for medicine delivery, by direct interview. The answers of the interviewees were transcribed word by word.

For data analysis, the technique of content analysis was used^10^. Meaning units were organized from the criterion of similarity of meaning. A mixed model was adopted to define the categories used in reports classification^10^. From a literature review and an initial reading of the reports, firstly we defined three categories or meaning units: conception of PS focused on medicines, conception of PS focused on activities destined to patients, and conception of PS as part of the management of health policies of a certain city.

In the following step, each report was read as many times as it was necessary to understand its meanings and carry out its classification. This operation, which was carried out by two researchers, led to the subdivision of previously defined categories, resulting in 12 categories containing the conceptions on PS. A set of reports with very distinct understandings was classified in the category “other”. The software SPSS®, version 21, complex samples analysis mode, was used to obtain conception frequencies, according to the interviewed subjects and Brazilian regions.

PNAUM was approved by the National Research Ethics Committee (Opinion no. 398.131/2013). The objectives of the research were explained to the interviewees, and they signed the informed consent form.

## RESULTS AND DISCUSSION

We interviewed 369 MSH (61.5% of the estimated sample); 507 RPS (84.5% of the estimated sample), and 1,139 professionals responsible for medicine delivery (83.6% of the estimated sample), among which 285 were PHARM, in the 272 cities of the research. Considering the insufficient percentage of answers given by secretaries, it was not possible to include them in the results by region.

Table 1 shows the sociodemographic profile of the interviewees. The male sex not only predominates among MSH, but also represents the highest percentages in the age groups over 31 years. Regarding education level, almost all RPS had higher education degree, while MSH had about 80%.

**Table 1:** Sociodemographic profile of those responsible for pharmaceutical services, pharmacists responsible for medicine dispensing in primary health care, and municipal secretaries of health, in Brazil. National Survey on Access, Use and Promotion of Rational Use of Medicines – Services, 2015.

Variable/subject	RPS		PHARM		MSH	
		
	%	IC95%	%	IC95%	%	IC95%
Sex						
Male	38.0	33.1–43.1	35.4	23.7–49.2	58.0	51.9–63.8
Female	62.0	56.9–66.9	64.6	50.8–76.3	42.0	36.2–48.1
Education level						
Some elementary school		––		––	0.6	0.2–2.5
Elementary school		––		––	1.1	0.4–3.0
Some high school					0.5	0.1–3.7
High school	1.2	0.6–2.1		––	16.1	12.2–20.9
Higher education	98.8	97.9–99.4	100.0	100.0–100.0	81.6	76.6–85.7
Age group (years)						
18 to 30	36.5	31.7–41.6	31.7	22.3–43.0	12.8	9.1–17.7
31 to 49	54.4	49.1–59.5	56.0	44.5–66.9	61.0	54.6–67.0
50 to 59	7.9	5.5–11.4	9.9	4.4–20.8	22.1	17.2–27.9
60 or more	1.2	0.4–3.5	2.4	1.0–5.5	4.2	2.4–7.0

RPS: those responsible for pharmaceutical services; PHARM: pharmacists responsible for medicine dispensing; MSH: Municipal Secretary of Health.

Source: PNAUM – Services, 2015.

Table 2 presents the conceptions on PS in Brazil, according to the understanding expressed by RPS, PHARM, and MSH. We observed differences between participants concerning the understanding of PS. Among RPS and PHARM, we highlight PS conceptions focused on guidance/information to users on the use of medicines, with or without references to their rational use, followed by conceptions focused on logistic cycle stages with guidance/information to users.

**Table 2:** Conceptions on pharmaceutical services in primary health care, according to the interviewed professionals. National Survey on Access, Use and Promotion of Rational Use of Medicines – Services, 2015.

Conceptions/Subjects	RPS	PHARM	MSH
		
	% (IC95%)	% (IC95%)	% (IC95%)
Focused on medicines:			
In stages of medicine logistic cycle	8.7 (6.2–12.0)	6.8 (3.4–13.2)	8.4 (5.5–12.7)
In stages of logistic cycle with guidance/information to the user of medicines	16.7 (13.2–20.9)	25.7 (14.9–0.40)	12.3 (8.7–17.0)
In the provision/offer of medicines	5.2 (3.3–8.3)	3.6 (1.6–7.8)	11.0 (7.5–15.7)
In controlled provision/offer of medicines	4.5 (2.8–7.1)	2.2 (0.3–13.6)	7.4 (4.7–11.5)
As part of the medical or pharmaceutical care procedure			
Activity performed by pharmacists in health care	1.5 (0.7–3.5)	0.4 (0.1–3.1)	0.6 (0.2–2.1)
As a complement or finalization of medical care, medicine dispensing, and user guidance	0.9 (0.3–2.7)	0.3 (0–2.1)	1.9 (0.7–4.6)
Associated with assistance to the poor			
As a way of assisting/supporting/helping deprived or low-income populations	0.2 (0–1.6)	––	4.0 (2.1–7.6)
Associated with the care/activities with the user of medicines			
Focused on the guidance/information to users on the use of medicines, with or without reference to their rational use	18.5 (14.8–22.8)	36.8 (26.1–48.9)	6.0 (3.6–9.7)
As a way of assisting/supporting/taking care of users/patients or population with guidance/information	8.1 (5.7–11.4)	11.2 (6.1–19.5)	13.9 (10.1–18.9)
Associated with care integrality	2.0 (0.9–4.3)	0.5 (0.1–3.3)	1.0 (0.4–2.7)
As part of the city’s health system management			
As a way of supporting/assisting management activities	2.3 (1.1–4.8)	––	4.7 (2.7–7.9)
As a way of organizing the services for the provision of medicines	2.4 (1.2–4.5)	––	6.4 (3.8–10.5)
Others	10.5 (7.8–14.0)	8.8 (4.6–16.1)	15.3 (11.3–20.5)
Did not know how to answer	0.4 (0.1–1.4)	1.3 (0.3–4.8)	3.0 (1.3–6.4)
Did not answer	18.1 (14.4–22.4)	2.5 (1.1–5.3)	4.2 (2.2–7.8)

RPS: those responsible for pharmaceutical services; PHARM: pharmacist responsible for medicine dispensing; MSH: Municipal Secretary of Health.

Source: PNAUM – Services, 2015.

The latter was also the second most frequent among MSH; in first place, there were conceptions on PS as a way of assisting/supporting/taking care of users or population. To following extracts illustrate these concepts:

*PS is a set of actions and information that must be provided to the user to leave no doubts on the use of medicines, thus providing their rational use (PHARM).*

*PS is responsible for medicine acquisition, maintenance, distribution, and guidance to users (RPS)*.

*PS is a set of actions in the context of SUS users that provide assistance, guidance, and qualified monitoring (MSH).*

Conceptions on PS regarding management and as a way of assisting/supporting/helping deprived or low-income populations were mainly expressed by MSH and were not found among pharmacists:

*PS are important to assist the health management of cities* (...) *(MSH)*.

*[They are] a health block within management, really important for the functioning of the Secretariat of Health, and serving as support (MSH)*.

*(...) supporting people in need by offering free medicines (MSH).*

*(...) aiding deprived people of a determined region (MSH)*.

The low percentages of PS conceptions associated with integrality of care among the interviewees called our attention, indicating the distancing of PS from other health actions, according to the perspectives of these subjects, and little understanding on PS as an essential component of SUS when it comes to integrality of health care. However, some interviewees reported understanding PS as a constituent of the health care process beyond medicines and perceptions that show some assimilation of the text of PNAF:

*I see PS as a segment of patients’ integral care, as part of the care process in our network that implies more than medicine acquisition and use (MSH).*

*They are actions that favor the prevention, rehabilitation, and protection of individual and collective health. They aim to promote the rational and correct use of medicines to improve the population’s quality of life (PHARM).*

*In my opinion, pharmaceutical services have the same objective as SUS. They are a set of actions aimed at health promotion, protection, and recovery. It is worth to highlight that their focus is ensuring the access to medicines and their rational use (…)* (RPS).

The interviewees reported the most diverse conceptions on PS; some of them defined them using only few words or only one, such as “excellent”; “essential”; “very important”; “the basis of care service (MSH)”; “useful, necessary, and economically beneficial to the city (RPS),” while others developed multiple meanings that did not allow us to categorize them within this study. We observed a wider variety of understandings of PS among MSH, followed by RPS and, finally, by the PHARM that are responsible for medicine delivery.

This diversity can be associated with characteristics of these subjects and their position on the health system: among MSH, we found the most diverse formations and education levels since elementary school; it was also among MSH that we found the highest percentage of interviewees that expressed not knowing what PS was. However, the highest percentage of those who did not answer the question occurred among RPS. It is possible that, because of their technical-administrative and political position within the municipal health system, they did not want to expose themselves or their perspectives on the matter without a formulation based on the PS national policy.

Furthermore, we highlight the limitations of the interviews, which could be: lack of disposition, motivation, or even inability of interviewees in answering appropriately; retention of information out of fear of having their identity revealed; or even provision of false answers^4^ ^,^ ^6^.

Table 3 presents conceptions on PS according to RPS, PHARM, and the regions. Besides varying between regions, the frequencies of conceptions also varied between the two interviewed groups.

**Table 3:** Frequency of conceptions of pharmaceutical services in primary health care, according to those responsible for pharmaceutical services and pharmacists responsible for medicine dispensing, according to Brazilian regions. National Survey on Access, Use and Promotion of Rational Use of Medicines – Services, 2015.

Conception/subject	North		Northeast		Midwest		Southeast		South	
				
	RPS	PHARM	RPS	PHARM	RPS	PHARM	RPS	PHARM	RPS	PHARM
Focused on medicines										
In stages of medicine logistic cycle	11.2	15.8	7.7	8.3	5.3	28.1	7.4	1.9	11.0	13.2
In stages of logistic cycle with guidance or information to medicine users	16.3	23.3	21.5	15.3	22.5	9.1	10.5	27.8	19.5	29.1
In provision or offer of medicines	2.3	4.4	8.8	3.4	––	8.1	5.3	3.9	2.1	1.9
In controlled provision or offer of medicines	4.6	––	2.6	4.2	––	––	5.2	3.6	6.0	––
As part of the medical or pharmaceutical care procedure										
Activity performed by pharmacists in health care	1.1	––	––	––	––	––	3.1	0.7	2.0	––
As a complement or finalization of medical care, medicine dispensing, and user guidance	2.6	––	1.3	––	––	––	1.1	––	––	1.9
Associated with assistance to the poor										
As a way of assisting/supporting/helping deprived or low-income populations	––	––	––	––	––	––	––	––	1.0	––
Associated with the care/activities with the user of medicines										
Focused on the guidance or information to users on the use of medicines, with or without reference to their rational use	20.6	43.9	15.1	19.0	32.0	40.6	17.8	35.1	23.0	42.2
As a way of assisting/supporting/taking care of users/patients or population with guidance/information	10.3	2.2	5.1	9.4	21.3	––	11.6	12.0	6.1	9.7
Associated with care integrality	1.1	––	2.5	2.1	––	––	3.2	0.9	––	––
As part of the city’s health system management										
As a way of supporting/assisting management activities	1.1	––	3.8	––	––	––	2.1	––	2.0	––
As way of organizing the services for the provision of medicines	6.0	––	2.6	3.1	––	––	2.1	––	1.0	––
Others	13.8	10.4	5.0	12.5	7.0	5.9	16.8	11.1	7.2	2.1
Did not know how to answer	1.1	––	––	1.0	1.1	8.1	––	0.9	1.0	––
Did not answer	7.7	––	24.0	21.8	10.7	––	13.7	2.3	18.1	

RPS: those responsible for pharmaceutical services; PHARM: pharmacists responsible for dispensing medicines.

Source: PNAUM – Services, 2015.

As expressed by two participants, conceptions centered on orientation or providing the patient with information for medicines use, with or without reference to their rational use, stood out in all regions, except in Northeast in the case of RPS.

Those responsible for pharmaceutical services, from all regions, were the only ones to express conceptions that associate medicine provision with the notion of control and management, which is possibly associated with their position in PS coordination. However, conceptions regarding health care integrality reached minimum percentages among these subjects and were only expressed by PHARM from the Southeast. As confirmed by active professionals of the public health system, these findings indicate that PS have not yet been incorporated as part of health care integrality, despite being a social right and a duty of the State.

The highest percentage of interviewees who reported not knowing what PS meant was among PHARM from the Northeast, region where we also noted the highest percentage of PHARM who did not answer the question.

It is worth to highlight that, in this study of exploratory character, it was not possible to deepen the analysis of the reports and their circumstances, such as the several contexts^10^ ^,^ ^11^, the production of statements on PS, sociopolitical contexts, advances in SUS implementation, and degree of institutionalization of PS in the cities of the interviewees.

We attempted to verify if the new curricular guidelines would have been influencing academic formation, and therefore changes in conceptions on PS. Table 4 presents the distribution of ideas expressed by PHARM of two age groups of up to 30 years old, considering that older pharmacists probably did not attend graduation courses that followed the *Diretrizes Curriculares Nacionais de Farmácia* (DCNF – National Curriculum Guidelines of Pharmacy), of 2002.

**Table 4:** Conceptions on pharmaceutical services in primary health care, according to pharmacists responsible for medicine dispensing, according to age group. National Survey on Access, Use and Promotion of Rational Use of Medicines – Services, 2015.

Conception	Age (%)		

	≤ 30 years	> 30 years	Total
Focused on medicines			
In stages of medicine logistic cycle	9.5	5.5	6.8
In stages of logistic cycle with guidance/information to the user of medicines	17.9	29.3	25.7
In the provision/offer of medicines	3.8	3.5	3.6
In controlled provision/offer of medicines	6.7	0.1	2.2
As part of the medical or pharmaceutical care procedure			
Activity performed by pharmacists in health care	––	0.6	0.4
As a complement or finalization of medical care, medicine dispensing, and user guidance		0.4	0.3
Associated with assistance to the poor			
As a way of assisting/supporting/helping deprived or low-income populations	––	––	––
Associated with the care/activities with the user of medicines			
Focused on the guidance/information to users on the use of medicines, with or without reference to their rational use	37.5	36.4	36.8
As a way of assisting/supporting/taking care of users/patients or population with guidance/information	9.8	11.8	11.2
Associated with care integrality		0.8	0.5
As part of the city’s health system management			
As a way of supporting/assisting management activities	––	––	––
As way of organizing the services for the provision of medicines	––	––	––
Others	7.4	9.5	8.8
Did not know how to answer	2.2	0.9	1.3
Did not answer	5.3	1.2	2.5

Source: PNAUM – Services, 2015.

We observed a similarity in the distribution of the most frequent conceptions in these two groups and a tendency to higher frequency of conceptions on technical and administrative activities in younger pharmacists. Among the latter, we did not find conceptions on PS associated with integrality of care, although they had occurred in small percentages among those with more than 30 years.

Within the limits of this study, we could not develop this question any further; however, our findings indicate that changes in professional formation introduced by the DCNF can be faster or slower depending on the changes in political-pedagogical processes and their association with the organization of services, which also require changes to meet the expectations within health policies.

Nicoline & Vieira^16^, when discussing the perspectives and challenges on pharmaceutical education concerning the PS category, consider the DCNF as wide and generic and, therefore, opened to several interpretations that do not ensure the formation of qualified pharmacists in the context of medicines and PS. Moreover, part of the group under study realized a greater need for a closer association between professional formation and curricular contents on management and planning of pharmaceutical services, in addition to the development of adequate methodological teaching strategies^16^.

To synthesize the data, we gathered PS conceptions in six macro-conceptions, presented in the Figure.

**Figure:**
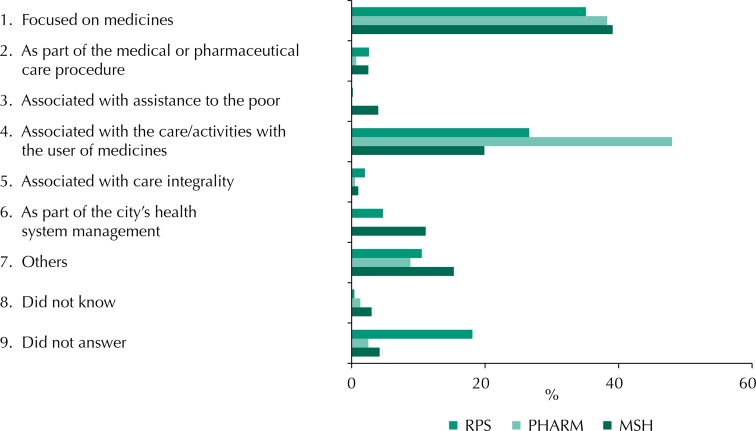
Macro-conceptions of pharmaceutical services according to those responsible for pharmaceutical services, pharmacists responsible for medicine dispensing in primary health care, and municipal secretaries of health, in Brazil. National Survey on Access, Use and Promotion of Rational Use of Medicines – Services, 2015.

RPS: those responsible for pharmaceutical services; PHARM: pharmacists responsible for medicine dispensing; MSH: municipal secretaries of health

Source: PNAUM – Services, 2015.

Our results show a high frequency of PS conceptions focused on medicines, as well as conceptions concerning the care/activities with users of medicines that predominate among pharmacists, indicating a tendency to the expansion of PS understanding.

This finding is relevant to more effective practices, given that conceptions focused on the programmatic aspect of PS, with emphasis on the logistic cycle, exert a limiting effect on the systemic and articulating character of PS rather than including the user of medicines as participant of the health care process and its final recipient. Thus, essential actions for the rational use of medicines and the technical bases of PS and their strategic position in health care are neglected, as well as other matters of SUS^3^ ^,^ ^17^.

The different reports expressed varied conceptions on PS and denoted a field under construction and consolidation of its services, practices, and references. Besides, they also can reflect the inclusion and improvement of activities in the work process of pharmacists, who, despite still being focused on the logistic cycle of medicines, also embrace PS concepts that refer to practices aimed at patient care. This could represent the expectations of subjects regarding changes in pharmaceutical practices within health services, in the current Brazilian context of PS reorientation.

According to Wells et al.^23^, pharmacists’ actions and beliefs on a service are key factors to analyze their performance and how services work in real life. The findings of this study point to a tendency of development in PS conceptions and also indicate that the process of consolidation of a robust pharmaceutical policy – as all public health policy should be – involves a complex process of development, implementation, and monitoring of the several involved aspects.

However, despite the favorable tendency identified, we still observed relevant frequencies of PS conceptions focused on the logistic cycle of medicines, as well as conceptions that emphasize guidance/information activities on the use of medicines. According to Perini^17^, this tendency of understanding PS by only focusing on logistics is very limiting as it hinders the comprehension of PS as part of care integrality.

As pointed out by Santos^19^, the discourse inherent in the systemic approach of PS refers to a set of articulated or sequential practices rather than health integrality structured by coordination, cooperation, and collaboration actions, as in the model proposed by Bradley, Ashcroft, and Noyce^5^.

Furthermore, the lack of understanding of PS as part of the management of the health system among PHARM suggests the limited experience of these professionals with managing functions, compromising the conduction and maintenance of necessary changes regarding organization. We observed an inverse tendency among MSH, which is probably due to their involvement with the health service patients’ demands, such as care, access, and benefits.

In addition, we can also consider that the concept of PS is still under construction, as it can be seen in the terminology adopted in the Federal Act no. 3,916, of October 30, 1998, which approves the *Política Nacional de Medicamentos* (PNM – National Drug Policy), and in the principles and strategic axes that should guide this segment of SUS, according to article number 1, subsection III, of the National Health Council resolution no. 338, of May 3, 2004, which approves PNAF^13^.

Vigotski^22^, in his experimental investigation, showed that the functional employment of words acts towards the active guiding of comprehension and abstraction, both essential in the process of concept formation. The organization of experiences assumes the ways followed in concept formation, i.e., the exercise of understanding words is not static and isolated, but it communicates with the process of thinking.

Developing concepts or acquiring meanings from words result from intense and complex activities that include the association of the word with the object and reality. According to Foucault^8^ (p. 55), “speeches are practices that systematically form the objects about which they speak.” That is why it is reasonable to state the formulation of concepts is a social construct capable of producing meanings and of central role in consciousness development. Language is a practical conscience^18^ that not only illustrates conflicts or domination systems, but also is the reason why we have something to fight for^8^.

## FINAL CONSIDERATIONS

Given the results, it is possible to conclude that the variety of conceptions on PS reflects an ongoing movement in the process of PS reorientation. We highlight that this movement occurs in different countries. In Brazil, both the National Drug Policy and PNAF have brought advances, establishing, among their guidelines, the reorientation of PS and the care to people, respectively. The reorientation process of PS in Brazil and other countries starts to take form with the gradual change from its technical paradigm (medicines) to an approach more focused on user-citizen patients^7^. Thus, pharmaceutical policies have been encouraging pharmacists to adopt a more multidisciplinary and collaborative attitude to achieve health care purposes.

The multiple understandings of PS found in the reports of strategic subjects comprise not only the meaning of the practices^9^ ^,^ ^11^, but also expose meaning disputes that exist in the area, which involve medicine, a complex object that produces meaning that is, at the same time, product, social good, health technology, and work environment.

There is need for theoretical and empirical studies on the subject; we suggest the deepening of the analysis of the subjects’ reports, considering their several contexts, and the expanded reflection with the distinct subjects involved, including citizens-users of health services, on PS and the space of medicine in health practices. The several conceptions of PS found reveal the aspects of a field under organization and consolidation of its services and practices, and also of its theoretical-conceptual references, showing a tendency of reversal logic based on a more procedural conception, focused on the medicine, towards one more focused on citizens. However, this is a gradual phenomenon. After all, this is a social, historical process that approaches subjectivities, worldviews, and ideologies that transcend the legal, technical-administrative, and logistic arrangements of the health system.

## Footnotes

a Ministério da Saúde (BR), Secretaria de Ciências, Tecnologia e Insumos Estratégicos. Departamento de Assistência Farmacêutica. Seminário de apresentação de resultados da PNAUM. Brasília (DF); 2015.
